# Modulation of the Acetylcholine Receptor Clustering Pathway Improves Neuromuscular Junction Structure and Muscle Strength in a Mouse Model of Congenital Myasthenic Syndrome

**DOI:** 10.3389/fnmol.2020.594220

**Published:** 2020-12-17

**Authors:** Sally Spendiff, Rachel Howarth, Grace McMacken, Tracey Davey, Kaitlyn Quinlan, Emily O'Connor, Clarke Slater, Stefan Hettwer, Armin Mäder, Andreas Roos, Rita Horvath, Hanns Lochmüller

**Affiliations:** ^1^Children's Hospital of Eastern Ontario Research Institute, Ottawa, ON, Canada; ^2^Institute of Neuroscience, Newcastle University, Newcastle upon Tyne, United Kingdom; ^3^Department of Neurosciences, Royal Victoria Hospital, Belfast, United Kingdom; ^4^Brain and Mind Research Institute, University of Ottawa, Ottawa, ON, Canada; ^5^Neurotune AG, Schlieren, Zurich; ^6^Department of Paediatric Neurology, University Hospital Essen, University of Duisburg-Essen, Essen, Germany; ^7^Department of Clinical Neurosciences, University of Cambridge, Cambridge, United Kingdom; ^8^Department of Neuropediatrics and Muscle Disorders, Medical Center – University of Freiburg, Freiburg, Germany; ^9^Centro Nacional de Análisis Genómico (CNAG-CRG), Center for Genomic Regulation, Barcelona Institute of Science and Technology (BIST), Barcelona, Spain

**Keywords:** neuromuscular junction, congenital myasthenia syndromes (CMS), therapies, neuromuscular disease (NMD), preclinical “*in vivo*” study, animal model, neuromuscular disorders

## Abstract

**Introduction:** Congenital myasthenic syndromes (CMS) are a diverse group of inherited neuromuscular disorders characterized by a failure of synaptic transmission at the neuromuscular junction (NMJ). CMS often present early with fatigable weakness and can be fatal through respiratory complications. The *AGRN* gene is one of over 30 genes known to harbor mutations causative for CMS. In this study, we aimed to determine if a compound (NT1654), developed to stimulate the acetylcholine receptor (AChR) clustering pathway, would benefit a mouse model of CMS caused by a loss-of-function mutation in *Agrn* (*Agrn*^nmf380^ mouse).

**Methods:** *Agrn*^nmf380^ mice received an injection of either NT1654 or vehicle compound daily, with wild-type litter mates used for comparison. Animals were weighed daily and underwent grip strength assessments. After 30 days of treatment animals were sacrificed, and muscles collected. Investigations into NMJ and muscle morphology were performed on collected tissue.

**Results:** While minimal improvements in NMJ ultrastructure were observed with electron microscopy, gross NMJ structure analysis using fluorescent labelling and confocal microscopy revealed extensive postsynaptic improvements in *Agrn*^nmf380^ mice with NT1654 administration, with variables frequently returning to wild type levels. An improvement in muscle weight and myofiber characteristics helped increase forelimb grip strength and body weight.

**Conclusions:** We conclude that NT1654 restores NMJ postsynaptic structure and improves muscle strength through normalization of muscle fiber composition and the prevention of atrophy. We hypothesize this occurs through the AChR clustering pathway in *Agrn*^nmf380^ mice. Future studies should investigate if this may represent a viable treatment option for patients with CMS, especially those with mutations in proteins of the AChR clustering pathway.

## Introduction

Proper functioning of the neuromuscular junction (NMJ) relies on efficient signaling to the muscle. This requires structural integrity of the NMJ which is mediated by a process that involves clustering of acetylcholine receptors (AChRs) at the postsynaptic membrane. This is achieved in part through the AGRIN/LRP4/MuSK pathway (Supplementary Figure 1). AGRIN is synthesized by motor neurons, muscle cells, and other non-neuronal cells, but it is only that transported from the motor neurons to the axon terminals that is comprised of the domains needed for AChR clustering (Bezakova et al., 2001). Neural AGRIN (z type generated by alternative splicing) released by motor nerves, binds to low-density lipoprotein receptor-related protein 4 (LRP4) which causes phosphorylation of muscle specific kinase (MuSK) (Kim et al., 2008). Once phosphorylated, MuSK recruits downstream of tyrosine kinase 7 (DOK7), which stimulates further MuSK phosphorylation (Burden et al., 2013). This causes receptor associated-protein of the synapse (RAPSYN) to form complexes with AChRs and help with their insertion into the postsynaptic membrane and anchoring to the cytoskeleton (Gervásio and Phillips, 2005). This pathway is important in both NMJ development and maintenance (Bezakova et al., 2001; Tezuka et al., 2014).

The congenital myasthenic syndromes (CMS) are caused by mutations in genes important for neuromuscular transmission. These mutations can be presynaptic, postsynaptic, in the synaptic cleft, or even in ubiquitously expressed proteins. CMS lead to disabling fatigable muscle weakness and can be fatal due to respiratory muscle weakness. Mutations in over 30 genes are known to be causative for CMS, and at least 16 have been identified in the *AGRN* gene (Finsterer, 2019; Ohkawara et al., 2020). *AGRN* mutations can impact AChR clustering by decreasing the phosphorylation of MuSK, degrading secreted neural AGRIN, and impairing the anchoring of AGRIN to the sarcolemma (Ohkawara et al., 2020). Patients with *AGRN* mutations present with exercise-induced proximal or distal muscle weakness, and varying degrees of ptosis, ophthalmoplegia, facial weakness, and respiratory muscle weakness. Neurophysiological tests typically reveal a decrement in compound muscle action potential on repetitive nerve stimulation, and muscle biopsies may show a myofibre Type I (TI) predominance, atrophy of Type II myofibers, an increase in small angular myofibers, and abnormal NMJ morphology (Huze et al., 2009; Maselli et al., 2012; Nicole et al., 2014).

Since the AGRIN protein is a key component of the AChR clustering pathway, it is possible that its manipulation could ameliorate the CMS phenotype. NT1654 is a 44 KDa C-Terminal neurotrypsin resistant fragment of mouse AGRIN (Hettwer et al., 2014) (Supplementary Figure 1). It has previously been used to ameliorate the NMJ pathology in rodent models of spinal muscular atrophy (Boido et al., 2018) and myasthenia gravis (Li et al., 2018), and in a zebrafish model of presynaptic CMS caused by mutations in *MYO9A* (O'Connor et al., 2018).

The aim of this study was to determine if NT1654 would benefit a mouse model of CMS caused by *Agrn* gene mutations. To answer this question, we used the *Agrn*^nmf380^ mouse generated through N-ethyl N-nitrosourea (ENU) chemical mutagenesis that induced a mutation in *Agrn* (nmf380-F1061S), resulting in a partial loss of function of the protein. Mice homozygous for the mutation are smaller than littermates, display poor hindlimb motor control and atrophy, and typically die within a few weeks to months of birth. They also have an increase in TI myofibers and abnormal NMJ morphology, closely recapitulating the human form of the disease (Bogdanik and Burgess, 2011).

## Materials and Methods

### Animal Husbandry

The *Agrn*^nmf380^ mouse with a point mutation in the *Agrn* gene on the C57BL/6J background was obtained from the Burgess lab (Bogdanik and Burgess, 2011). All animal procedures were performed in accordance with the Animals Scientific Procedures Act of 1986 under project license 70/8538. Animals were housed under 12 h light/dark cycles in the Functional Genomics Unit, at Newcastle University, and had access to standard chow and water *ad libitum*. Since *Agrn*^nmf380^ mice had hindlimb wasting, soaked diet was always available on the cage floor. Animals were weighed and monitored daily and any animal found to have lost >17% of body weight or showing a severe phenotype was humanely culled.

### Treatment Protocol and Tissue Collection

Animals were assigned at postnatal day 5 (P5) to either a drug treatment (NT) or vehicle control (Veh) group, with wild type (WT) litter mates being used for comparison. *Agrn*^nmf380^ mice assigned to the NT group received a daily sub-cutaneous injection (scruff of the neck) of NT1654 (Neurotune AG), a modified form of AGRIN, at 10 mg/kg. The drug was carried in phosphate-buffered saline (PBS) so *Agrn*^nmf380^ mice assigned to the Veh group received a daily injection of PBS at a corresponding volume. Animals were injected for 30 days and culled at the end of the study by cervical dislocation and tissues harvested and weighed. A pilot study previously examined the effects of administering 2, 5, and 10 mg/kg of NT1654 to WT and *Agrn*^nmf380^ animals. No deleterious effects in terms of body weight, liver enzymes, or liver and kidney histology were observed at any drug concentration (data not shown), so the highest concentration (10 mg/kg) of the drug was used. This was also consistent with previous rodent trials of this compound that produced positive results (Hettwer et al., 2014; Boido et al., 2018).

### Muscle Strength Assessments

Animals underwent three strength assessments. The first was a hindlimb suspension test designed for neonates at P7. Following this at P22-24 and P33-35 grip strength was assessed using a Grip Strength meter with grid attachment (Bioseb), both according to established protocols (Willmann et al., 2011). Both tests were performed in a room separate to the holding room with only the tester present to minimize disturbance for the animals. For the hindlimb suspension test, neonates were suspended face down by their hindlimbs inside a 50 ml falcon tube containing bedding packed into the bottom of the tube. Measurements of hanging time prior to falling, number of pulls, and a hindlimb suspension score (HLS) based on the evaluation of hindlimb spread, were recorded. The average of three attempts was taken. For grip strength assessment the mouse was held firmly by the tail and allowed to grab the grid with either its front or all four paws and then pulled back in the horizontal plane until the animal released its grip. Animals were tested six times (3 × forelimb, 3 × fore and hindlimb) in a session with a break of at least 1 min between tests. No decrease in grip strength was noted across the six tests performed during the testing session. The force recorded on the meter was noted and the mouse returned to its home cage. Experiments were conducted in a blinded fashion.

### Myofiber Characteristics

Labelling of myosin heavy chains (myofiber type) and laminin was performed as previously described (Aare et al., 2016). Muscles for labelling were mounted on cork discs using optimal cutting temperature compound and then frozen in liquid nitrogen pre-cooled isopentane before being stored at −80°C. Transverse 10 μm sections of the soleus muscle were cut using a Microm HM 500 cryostat and labelled for myosin heavy Chains (MHC) and laminin. Muscle was first washed 1 × 5 min in PBS, then blocked in 10% normal goat serum (NGS) for 1 h at room temperature (RT). Primary antibodies were purchased from Developmental Studies Hybridoma Bank; BA-F8 (mouse anti-MHC1 IgG2b, 1:25), Sc-71 (mouse anti-MHC2a IgG1, 1:200), and BF-F3 (mouse anti-MHC2b 1:200) were applied to one section, and on a second serial section 6H1 (mouse anti-MHC2 × IgGM, 1:25) was labelled. Both sections were also reacted against rabbit anti-laminin IgG (1:750, Millipore-Sigma). Muscles were incubated for 1 h (RT) and then washed 3 × 5 min in PBS. Secondary antibodies (ThermoFisher Scientific): Alexa Fluor 350 IgG2b (y2b) goat anti-mouse (A-21140, 1:500), Alexa Fluor 594 IgG1 (y1) goat anti-mouse (A-21125, 1:100), Alexa Fluor 488 IgM goat anti-mouse (A-21042, 1:500), and Alexa Fluor 488 IgG goat anti-rabbit (A-11008, 1:500) were applied for 1 h (RT). Following this, sections were given a final wash of 3 × 5 min and mounted using Vectashield hardset mounting medium (Vector Laboratories). Tiled images of the whole muscle were captured on a Zeiss Axio Imager microscope with Zen software and analyzed using FIJI (Schindelin et al., 2012) by an investigator blinded to the experimental group. Transverse myofibers were traced, and the MHC type noted. The percentage of each MHC type was noted for each animal and then these data used for statistics. Fiber type grouping was recorded through the identification of ‘enclosed myofibers’, i. e., those that were completely surrounded by myofibers of the same MHC type. The number of enclosed fibers was then expressed as a percentage of the total fibers recorded for that animal.

Hematoxylin and Eosin (H & E) staining was performed on transverse sections from the soleus muscle. Sections were initially rinsed in tap water, then placed in Myer's Hematoxylin (Millipore-Sigma) for 1 min. They were then rinsed in tap water until the water ran clear and placed in eosin (Millipore-Sigma) for 30 s. They were again rinsed in tap water, dehydrated through a graded ethanol series (75, 95, 100% × 2), cleared with two changes of xylene and mounted using DPX Mountant for Histology (Millipore-Sigma). Images were captured using a Mbf Biosciences Tissue Scope.

### NMJ Labelling, Capturing, and Analysis

NMJ labelling was performed on soleus muscle using previously published protocols (Cipriani et al., 2018). Muscles were washed in ice–cold PBS for 2 × 10 min and then separated out into small bundles of about five fibres using tweezers under a stereo-microscope. They were fixed overnight at 4°C in 2% paraformaldehyde (PFA). The following morning, they were washed 2 × 1 h with ice–cold PBS to remove the PFA. They were treated for 10 min with Analar Ethanol followed by 10 min with Analar Methanol, both at −20°C. Tissues were then incubated with blocking/permeabilization solution (5% horse serum, 5% BSA, 2% Triton X-100 in PBS) for 4 h at RT with gentle agitation. Muscle bundles were incubated with antibodies, diluted in blocking buffer without triton, against neurofilament (mouse monoclonal IgG1, Cell Signalling, 1:100) and synaptophysin (rabbit polyclonal, ThermoFisherScientific, 1:100), overnight at 4°C with agitation and then for a further 2 h at RT the next morning. Muscles were then washed in blocking buffer 4 × 1 h at RT. They were incubated with Alexa 488-Conjugated α-Bungarotoxin (ThermoFisherScientific 1:250), Alexa Fluor 594 goat anti-rabbit IgG, and Alexa Fluor 594 goat anti-mouse IgG1 (ThermoFisher 1:200) for 4 h at RT. Samples were washed 4 × 1 h in PBS and then mounted using Vectashield hardset mounting medium. Images were captured using a Nikon A1R laser scanning confocal microscope using NIS-elements AR 4.20.02 software. Parameters were kept constant for all samples and Z-stack images were acquired with a ×63 oil immersion objective at 2 μm intervals.

Analysis was performed blinded according to the NMJ_Morph protocol (Jones et al., 2016) developed using FIJI (Schindelin et al., 2012). To set the threshold, a maximum intensity projection was used as a reference while adjusting the threshold for the image so that it best matched the maximum intensity projection. In addition to the 19 variables calculated as part of the NMJ_Morph package, we also quantified the NMJs that completely lacked either synaptophysin or α-bungarotoxin staining. All variables were calculated from each NMJ with the ranges of NMJs studied were 16 to 44 for WT, 2 to 17 for PBS and 13 to 43 for NT animals. While this was below the recommended number of 30 suggested in the protocol, the investigator endeavored to capture all NMJs in each muscle preparation.

### Electron Microscopy

Electron microscopy (EM) of the intercostal muscles from male mice was performed by the Electron Microscopy Research Services at Newcastle University. Tissues were fixed in 2% buffered glutaraldehyde, osmicated in 1% phosphate-buffered osmium tetroxyde, then dehydrated and embedded in epoxy resin. One micro meter semi-thin sections were stained with toluidine blue and then ultrathin sections were contrasted with uranyl acetate and lead citrate. EM images were captured using a CM10 transmission electron microscopy (Philips, Amsterdam, The Netherlands). Images were analysed using FIJI (Schindelin et al., 2012) with the observer blinded to the experimental group. Measurements were made of a number of variables according to previously published protocols (Slater et al., 1992; Wood and Slater, 1997), along with measurements of the number of secondary synaptic folds, their length, and number of branches, width of synaptic cleft, Schwann cell infiltration into the cleft, and the number of folds with clusters of synaptic vesicles opposite (Supplementary Figure 2).

### Statistics

Data were initially recorded using Microsoft Excel and then analysed using GraphPad Prism v 8.4.0. Data were checked for normality using the D'Agostino-Pearson test and the appropriate statistical analysis applied as described in the figure legend. Outliers were identified using the ROUT (Q = 1%) method. Data points that were found to be outliers were left in place but their presence in the data set highlighted in the figure legends. Normally distributed data are expressed as mean ± standard deviation (SD), while data not normally distributed are presented as median (25th, 75th percentile) number of NMJs analyzed. A *P* value of <0.05 was considered significant and values are given in the figure legend.

## Results

*Agrn*^nmf380^ animals were given daily injections from postnatal day 5 (P5) for 30 days with either 10 mg/kg of NT1654 (NT) or PBS (Veh), with a WT group also included for comparison. Data were collected from 9 WT (4M and 5F), 7 Veh (3M and 4F), and 7 NT (2M and 5F) animals. Seven animals (4 Veh and 3 NT) had to be humanely culled during the study due to weight loss of more than 17%, as per our animal welfare guidelines. This resulted in the average ages of the Veh and the NT animals being younger that WT at tissue harvest (WT 36.13 ± 0.83 days, Veh 24.57 ± 8.63 days, NT 27.29 ± 10.66 days) which may have influenced comparisons between WT and *Agrn*^nmf380^ animals for some variables. While experimental animals were obtained through Het × Het crosses, only 12.4% of homozygous animals were obtained, with the remaining 51.3% being heterozygous and 36.3% WT animals. As a result, we were unable to have equal numbers of males and females in each group and for some variables sex specific differences were noted, for example body weight and NMJ morphology. In these cases, the results from just a single sex have been presented (that for which a greater number were present) and this has been indicated in the figure legend.

### Body Weight

**Figure 1:**
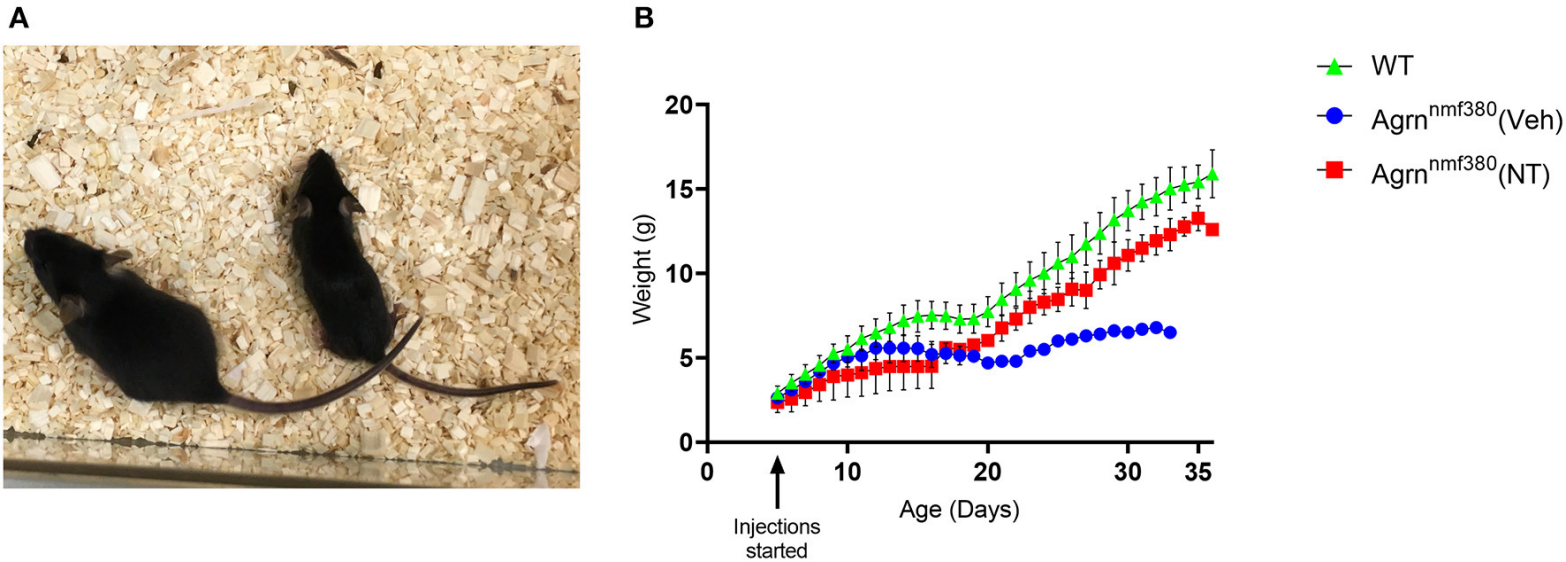
Body weight of female *Agrn*^nmf380^ animals treated with NT1654. Veh animals displayed hindlimb wasting, a slight curvature of the spine and were considerably smaller than their WT littermates at 31 days **(A)**. Administration of NT1654 partially rescued body weight in *Agrn*^nmf380^ animals **(B)**. Graph shows mean ± S.D, *N* = 5 WT, 4 Veh, 5 NT. 1 WT, 3 Veh, and 2 NT animals were culled due to weight loss before the end of the study, which will have impacted the mean and SD values in this graph.

Animals were weighed daily to determine the amount of drug to administer, as a health monitoring tool, and as an experimental variable. As previously noted (Bogdanik and Burgess, 2011), Veh animals were considerably smaller with hindlimb muscle wasting (Figure 1A) and spinal curvature. Their body weight was considerably reduced in comparison to WT littermates (Figure 1B and Supplemental Figure 3). Administration of NT1654 improved body weight though not back to WT levels. Interestingly, the body weight of the NT and Veh animals diverged around day P16-17, which is consistent with the previously observed complete breakdown in NMJ morphology by P18 in these animals (Bogdanik and Burgess, 2011). We did not observe any improvement in the spinal curvature.

### Muscle Strength

**Figure 2:**
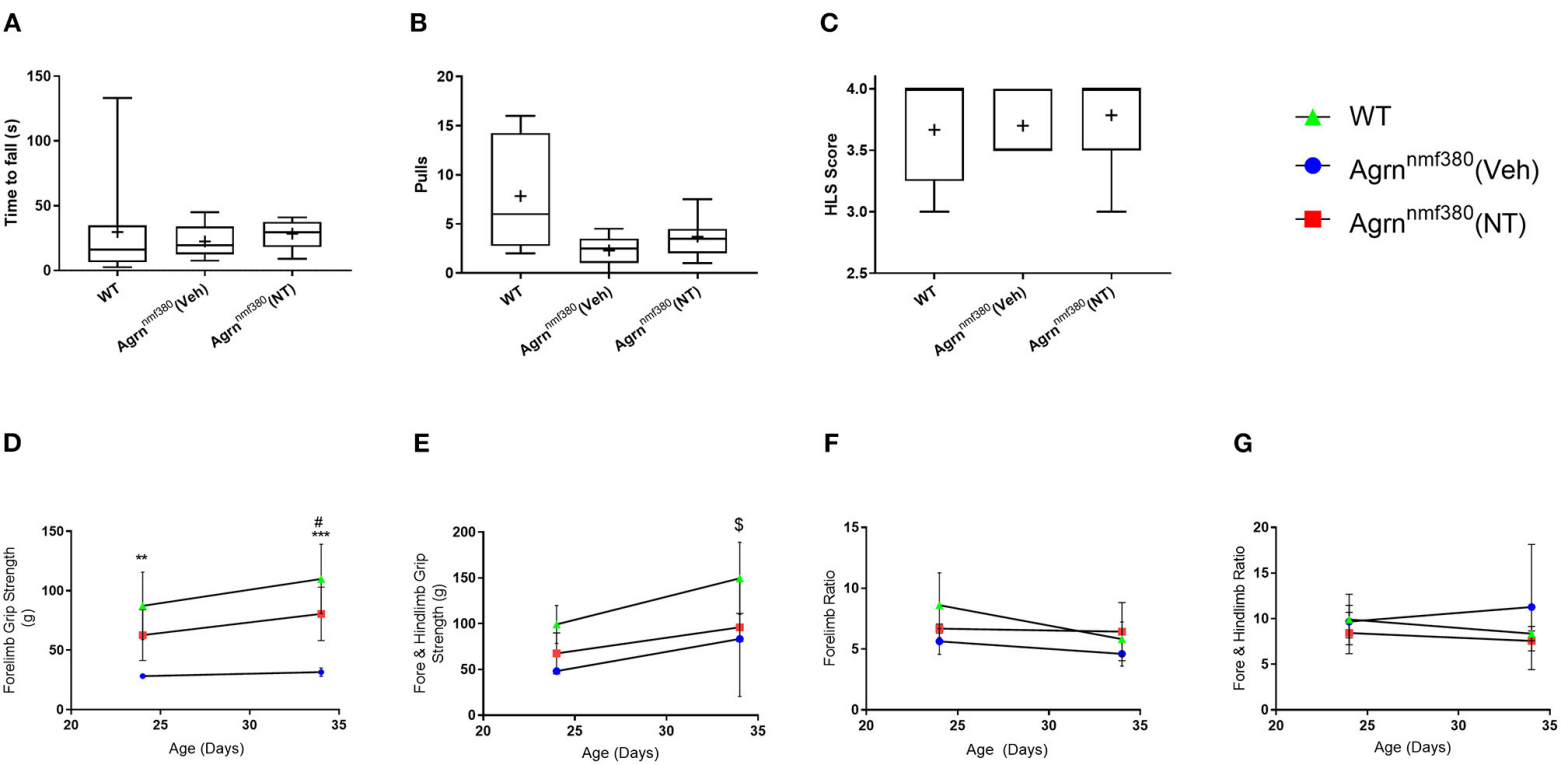
Strength assessment in WT, Veh, and NT male and female animals. **(A)** At P7 no significant difference was observed in seconds to fall (Kruskal–Wallis test followed by Dunn's multiple comparisons test). The value at 133 s was identified as an outlier but had no effect on the significance level of the data. **(B)** The number of pulls also did not vary considerably between groups (Kruskal–Wallis test followed by Dunn's multiple comparisons test). **(C)** There were no differences in the HLS scores between WT, Veh, and NT animals. While the NT scores of 3 and 3.5 were identified as data outliers they did not influence the significance of the test (Kruskal–Wallis test followed by Dunn's multiple comparisons test). Box and whiskers plots show 5–95 percentile, with + indicating the mean. *N* = 9 WT, 5 Veh, 7 NT. **(D)** WT animals had significantly stronger forelimb grip strength than Veh animals during at P24 and P34. Treatment with NT1654 significantly improved forelimb grip strength over Veh animals at P34 (Mixed effect model followed by Sidak's multiple comparisons tests). **(E)** WT animals were significantly stronger than both Veh and NT animals following 30 days of treatment in terms of combined fore- and hindlimb grip strength (Mixed effect model followed by Sidak's multiple comparisons tests). Normalizing grip strength to body weight revealed that grip strength was dependent on mass for both fore **(F)** and combined fore and hindlimb strength **(G)** (Mixed effect model followed by Sidak's multiple comparisons tests). Graphs show mean ± SD. *N* = 9 WT, 3 Veh, 4 NT *Denotes significant difference between WT and Veh, *P < 0.05 **P < 0.005 ***P < 0.0005, ^#^Denotes significant difference between NT and Veh ^#^*P* < 0.05, ^$^denotes significant difference between WT and NT ^$^*P* < 0.05.

Muscle strength was measured using a hindlimb suspension test (tube test) at P7 and by a grip strength assessment using a grip strength meter at P24 and at P34. At P7 there was no significant difference in hang time between WT, Veh, or NT animals (Figure 2A). The number of pulls the mice attempted was also recorded (Figure 2B). WT animals pulled up more than either the Veh or NT animals, but again there was no significant difference between groups. The hind limb suspension (HLS) score is denoted by the position of the legs on the tube with a four denoting a raised tail and legs spread wide on the tube and a one being given for legs completely together and a lowered tail (Willmann et al., 2011). There were no significant differences between groups in HLS scores (Figure 2C). This was consistent with NMJs in the limbs of the *Agrn*^nmf380^ mice having a ‘normal’ innervation and size at day P9 (Bogdanik and Burgess, 2011).

Around P24, differences between WT and Veh animals in terms of forelimb grip strength became apparent. WT animals were more than twice as strong as Veh animals, with no significant differences between Veh and NT animals. By P34 WT animals remained significantly stronger than Veh animals, and interestingly treatment with NT1654 made NT animals over twice as strong as Veh animals. While NT animals were never quite as strong as WT animals, there was never any significant difference noted between them at either time point (Figure 2D). In addition to forelimb grip strength we also measured combined fore- and hindlimb grip strength by holding the mouse in such a way to allow it to grip the grid with all four paws. At P24 there was no significant difference in grip strength between the three animal groups (Figure 2E). By P34 of the study WT animals were significantly stronger than both Veh and NT animals. Unlike forelimb grip strength, NT animals were not significantly stronger than Veh animals following 30 days of treatment. *Agrn*^nmf380^ mice display extensive hindlimb wasting (Figure 1A) and it is possible that this was not rescuable by the NT1654 treatment. By dividing the force generated in grams (g) by the body weight of the animals it is possible to determine the force ratio. For both fore- and combined fore-/hindlimb grip strength, there were no differences at any time point in force generated (Figures 2F,G). This could suggest that differences in force were attributable to an increase in muscle mass.

### Muscle Weights

**Figure 3:**
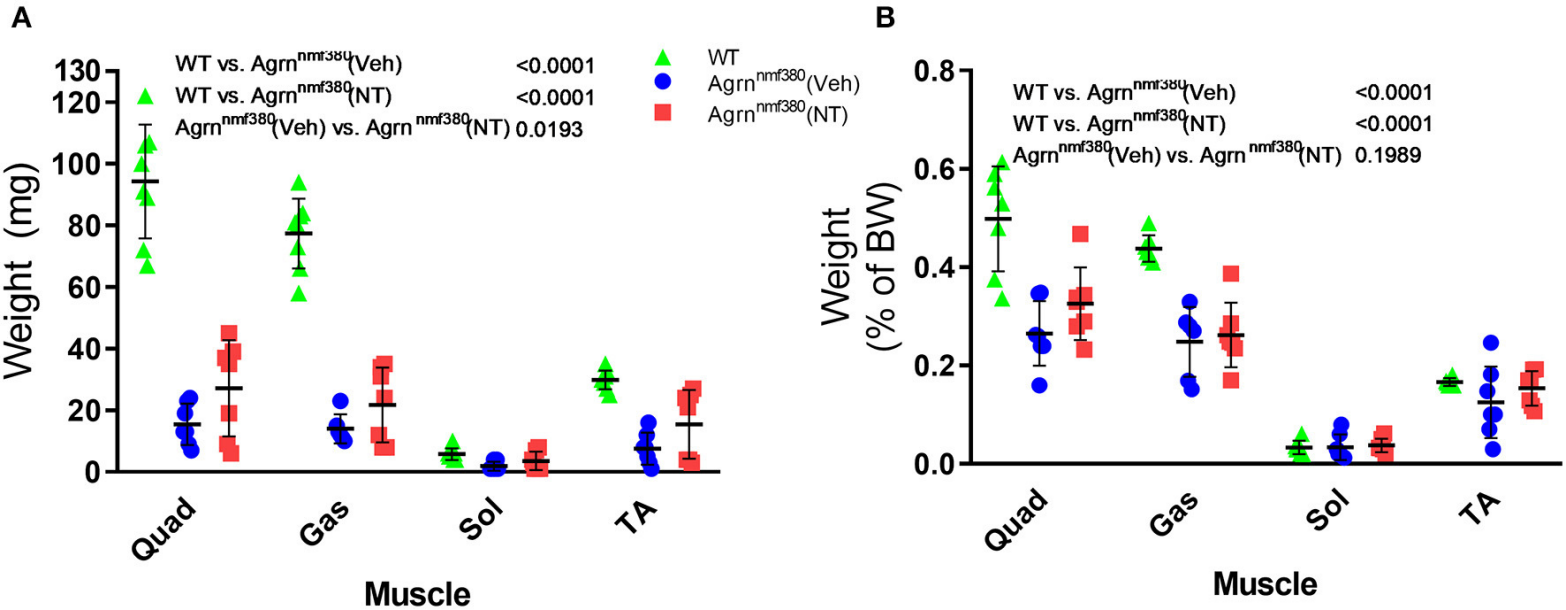
Muscle harvest weight in male and female animals. Hindlimb muscles were blotted dry and weighed following excision. Treatment with NT1654 partially rescued muscle weight although not back to WT levels (2-way ANOVA followed by Tukey's multiple comparison tests) **(A)**. Following normalization to body weight NT animals no longer demonstrated a heavier muscle weight over Veh animals **(B)**. (2-way ANOVA followed by Tukey's multiple comparison tests) *N* = 8 WT, 7 Veh, 7 NT, Quad, quadriceps; Gas, gastrocnemius; Sol, Soleus; TA, Tibialis anterior.

As we hypothesized that the changes in absolute grip strength were attributable to an increase in muscle mass, we weighed the muscles during harvest (Supplementary Table 1 and Figure 3). WT animals had significantly heavier absolute muscle weight than Veh animals (*P* < 0.0001). The reduction of muscle mass in Veh animals was partially prevented by drug administration in NT animals (Figure 3A). While NT animals had significantly heavier muscles than Veh animals (*P* = 0.0193) they did not fully recover and remained significantly lighter than WT animals (*P* < 0.0001). When expressed as a percentage of body weight (Figure 3B) WT animals continued to have significantly heavier muscles than Veh and NT animals (*P* < 0.0001). However, NT animals no longer had a heavier muscle weight than Veh.

### Myofibre Morphology, Myosin Heavy Chain (MHC) Type, and Myofiber Type Grouping

**Figure 4:**
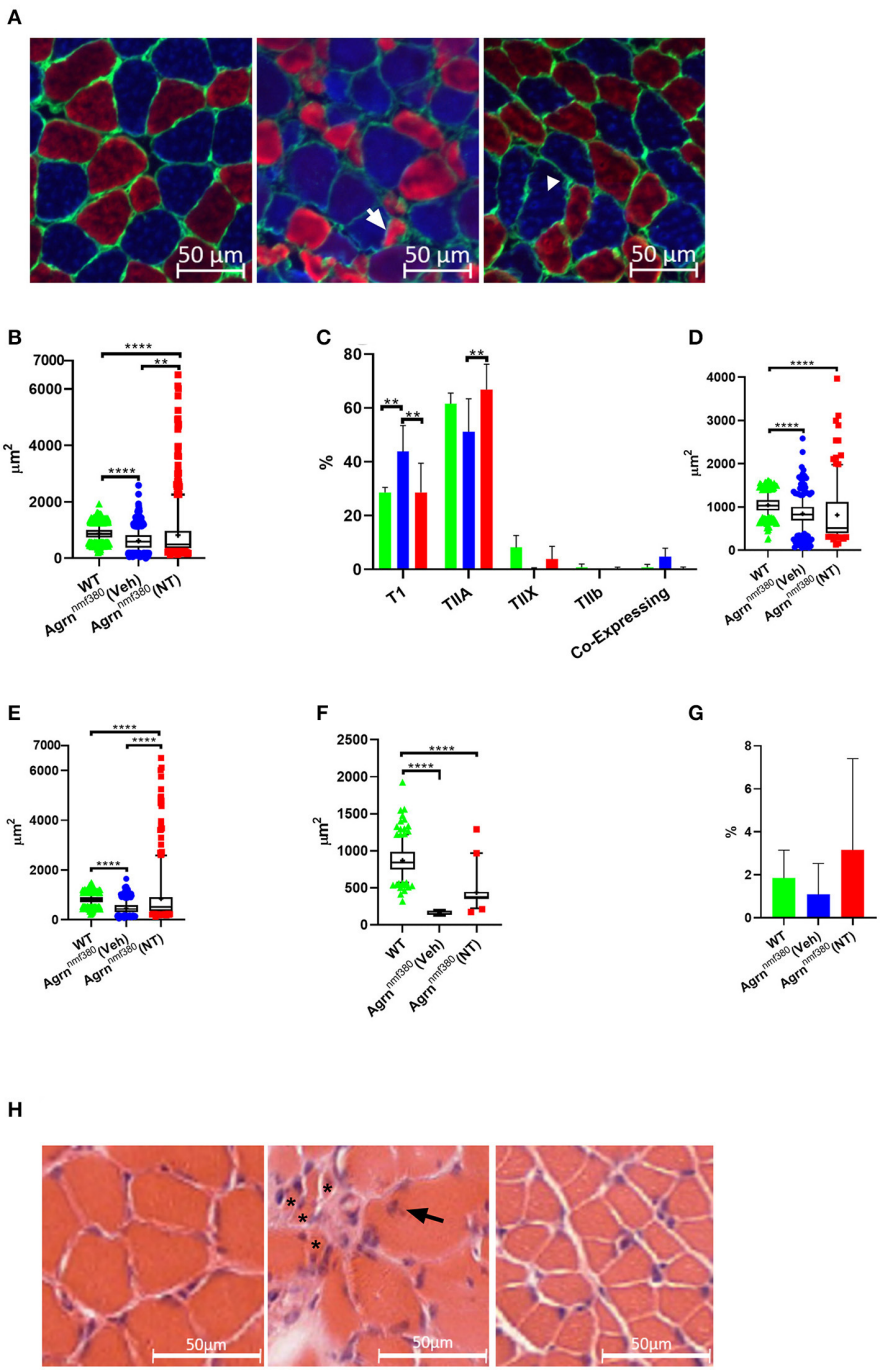
Myofiber morphology and type in WT, Veh, and NT female animals. **(A)** Soleus muscle was labelled for MHC in WT (left), Veh (middle), and NT (right) animals, so that Type I myofibers = blue, Type IIa myofibers = red, Type IIb and Laminin = green. Type IIx myofibers were labelled on a serial section. Arrow indicates an atrophic TIIa fiber and the arrowhead indicates a hypotrophic TI fiber. **(B)** While Veh animals had significantly smaller myofibers than WT animals the fiber size was increased with NT1654 administration through a combination of a reduction in very small fibers and hypertrophy of other fibers (Kruskal–Wallis test followed by Dunn's multiple comparisons test). **(C)** Veh animals had an increased percentage of TI myofibers coupled with a decrease in TIIa/x myofibers, this was prevented with NT treatment (2-way ANOVA followed by Tukey's multiple comparison tests). Veh animals had smaller TI **(D)**, TIIa **(E)** and TIIx **(F)** myofibers than WT animals. TII myofiber atrophy was partially prevented with drug treatment in the NT animals (Kruskal–Wallis test followed by Dunn's multiple comparisons test). **(G)** There were no significant differences in the percentage of enclosed myofibres between groups. Box and whiskers plots show 5-95 percentile, with + indicating the mean, bar charts show mean + SD. (*N* = 4 WT, 3 Veh, 4 NT, **P < 0.005 ****P < 0.0001). **(H)** Transvers soleus muscle from WT (Left), Veh (middle), and NT (right) animals were imaged following H and E staining. In the Veh animals multiple very small atrophic myofibers (*) were visible, along with internalized nuclei (arrow).

Images were labelled for MHCs (Figure 4A) and analyzed using Fiji with the observer blinded to the experimental group. As expected following the muscle weight data, WT animals had a significantly larger (42%) myofiber area than Veh, which was then increased in NT animals so that there only remained a 7% difference in myofiber size between WT and NT animals. This increase came from a rescue of very small fibers and a hypertrophy of other fibers (Figures 4A,B). As previously reported in both patients (Huze et al., 2009; Maselli et al., 2012; Nicole et al., 2014) and *Agrn*^nmf380^ mice (Bogdanik and Burgess, 2011), Veh animals had a significantly higher percentage of TI myofibers than WT (Figure 4C). This increase in TI myofibres is coupled with a reduction in the percentage of TIIa and TIIx myofibers. This could be attributable to atrophy of TIIa and TIIx fibers or a fiber type shifting to an increasingly slow phenotype. Interestingly, treatment with NT1654 restored myofiber type proportions to WT levels, with NT mice demonstrating a similar TI myofiber percentage to WT animals, and an increase in TIIa myofibres when compared to Veh mice (Figure 4C).

To investigate the fiber type changes further, we analyzed myofiber size by type. TI myofibers of Veh animals were 81% the size of those from WT animals, however their TIIa and TIIx myofibers were only 58 and 19%, the size of WT animals respectively, suggesting a greater atrophy of TII myofibers over TI (Figures 4D–F, Supplementary Table 2). Treatment with NT1654 had no significant effect on TI myofiber size, although there did appear to be a small accumulation of fibers with a much larger cross-sectional area and slightly fewer with a very small cross-sectional area. Treatment partially rescued TIIa and TIIx myofiber size suggesting that NT1654 treatment reduced the TII myofiber atrophy seen in Veh animals, accounting for the normalization of the fiber type. MHC co-expressing myofibers, those displaying more than one MHC, are often used as an indicator of myofiber denervation (Aare et al., 2016). While Veh animals had higher levels of co-expressing myofibers than both WT and NT mice, this difference was not significant (Figure 4C). Another marker of denervation often observed in the muscle is that of myofiber type grouping, where a myofiber is entirely enclosed by myofibers of the same MHC. This is an indicator of myofiber reinnervation by a neighboring nerve following myofiber denervation. However, there was no significant difference in the number of enclosed myofibers between groups (Figure 4G).

A test for outliers suggested a number of values for the NT animals (71) were outliers, while the number of outliers in WT (1) and Veh (3) was considerably lower. A Barlett's test demonstrated that these groups had significantly different standard deviations (*P* < 0.0001), which is clearly visible in the long ‘tail’ of higher values for the NT animals (Figure 4B). These large myofibers could represent a response to the NT1654 treatment rather than being ‘true outliers’, especially as they were mostly accounted for by TIIa myofibers (53).

H & E staining in the Veh animals revealed a very non-uniform muscle pattern (Figure 4H) characterized by uneven fiber size including very small fibers, internalized nuclei, and fibrotic tissue. Fiber morphology appeared more uniform in the NT animals, although the fibers still appeared smaller.

### NMJ Structure

**Figure 5:**
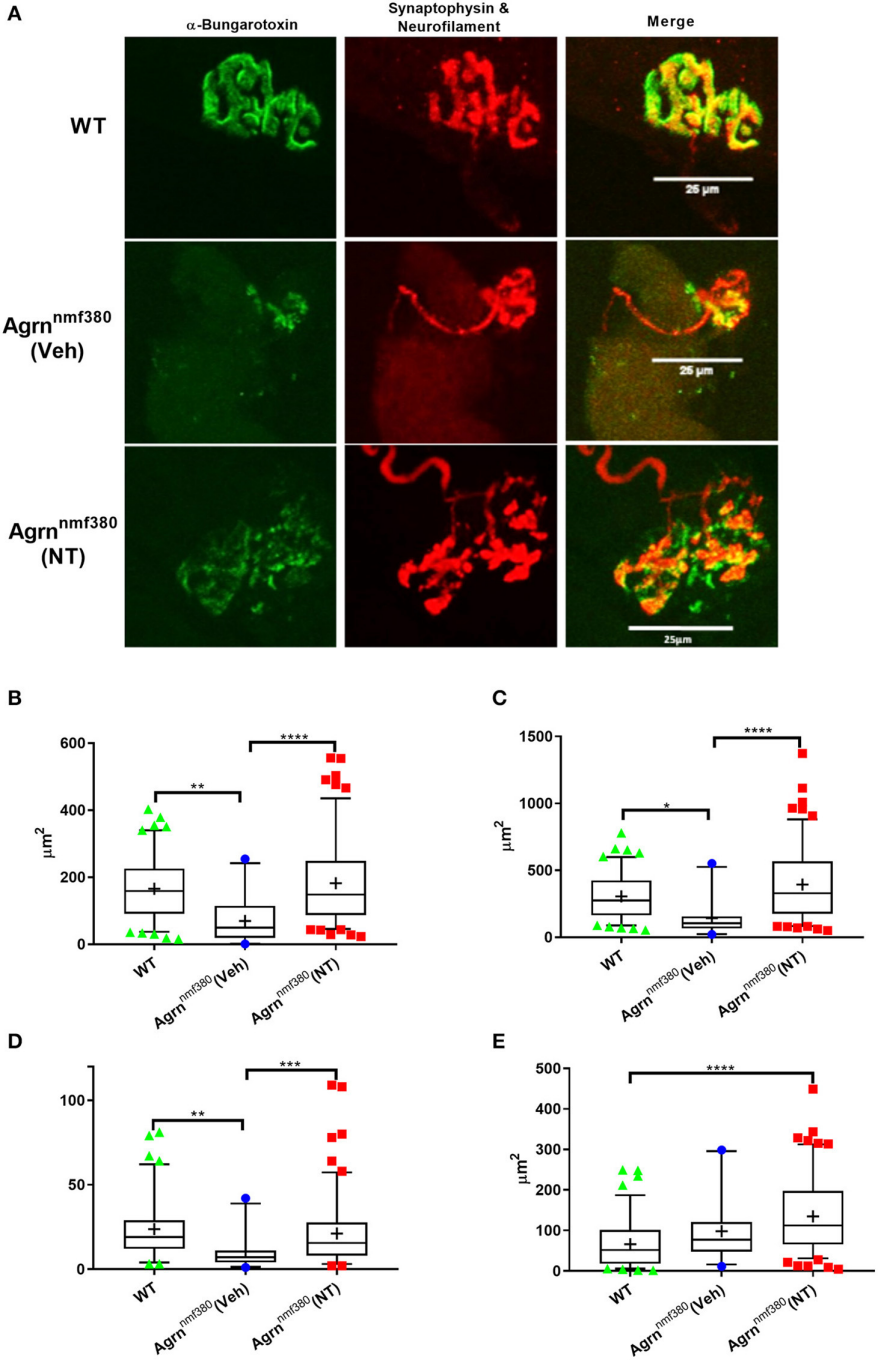
NMJ structure in WT, Veh, and NT female animals. **(A)** Whole muscle mounts of the soleus were labelled with α-bungarotoxin (green), neurofilament (red), and synaptophysin (red). Analysis was performed on maximum intensity projections as stipulated in the analysis protocol. Images shown have been adjusted for brightness and contrast to better demonstrate the image features, scale bar is 25μm. WT animals had larger AChR area **(B)**, endplate area **(C)**, and number of terminal branches **(D)** than Veh animals, which returned to WT levels in NT animals. The nerve terminal area was significantly larger in NT animals compared to WT **(E)**, despite this not being an impacted variable. Kruskal–Wallis test followed by Dunn's multiple comparisons test, Box and whiskers plots show 5–95 percentile, with + indicating the mean, *N* **=** 4 WT, 3 Veh, and 5 NT, **P < 0.005, ***P < 0.0005, ****P < 0.0001.

Abnormal NMJ morphology has been noted in both humans and mice with mutations in the *AGRN* gene (Bogdanik and Burgess, 2011; Nicole et al., 2014), this was evident in the current study (Figure 5A and Supplementary Table 3). We measured NMJ structure in the soleus muscle using antibodies against neurofilament and synaptophysin and using α-Bungarotoxin to label AChRs (Figure 5A) and then in the intercostal muscles with EM (Supplementary Figure 4).

In the soleus muscle postsynaptic and some presynaptic variables were impacted by the *Agrn* mutation (Supplementary Table 3), with Veh animals having a reduced AChR and endplate area (Figures 5B,C) as determined with α-Bungarotoxin staining. Treatment with NT1654 prevented these decreases to the point that NT animals were no longer significantly lower than WT animals. A number of NMJs in the Veh mice did not have any α-bungarotoxin labelling, indicating a lack of AChRs, this was never observed in the WT or NT animals (Supplementary Table 3). Unlike other studies that have investigated NMJ pathology, we did not observe any change in the degree of fragmentation in our mouse model of NMJ dysfunction (Supplementary Table 3). We also observed a number of changes in presynaptic measurements, despite the presynapse not being the primary target of the mutation or the treatment. The number of terminal branches was decreased in Veh animals compared to WT, but this did not occur in NT mice (Figure 5D). The nerve terminal area was significantly bigger in NT mice when compared to WT (Figure 5E), despite this not being an impacted variable in the Veh animals, demonstrating the remarkable plasticity of the NMJ.

As with myofiber area, the variance of morphological parameters in NT animals was considerably greater than in either the WT or Veh models (Figures 5B–E and Supplementary Table 3). Similar to Figures 4B,D,E, key NMJ variables had a long tail of large values. AChR area (Figure 5B) and nerve terminal area (Figure 5E) both had unequal variances in their distributions (Bartletts test, *P* < 0.0005), with NT animals showing an elongated tail towards the larger areas. Unfortunately, we were unable to measure the size of the myofibers on which we measured these NMJ morphological parameters, so we are unable to determine if these larger NMJs were present on the larger myofibers.

In the intercostal muscles of male mice, a visual inspection of electron-micrographs appeared to show disruption at the NMJ (Supplementary Figure 4), this was not reflected in the measurements recorded. The only difference between groups was an increase in the length of the postsynaptic folds (CFL) in the Veh animals which returned to WT levels in NT animals (Supplementary Table 4). An increase in fold length was also observed in a previous study of *Agrn*^nmf380^ animals (Bogdanik and Burgess, 2011). No differences were noted in the length of the presynaptic terminal in contact with the myofiber (PreL), the total length of the postsynaptic membrane (FoldL), the number of folds (NF), or in derived variables like the branching index (number of branches divided by NF), or the occupancy, which is a measure of the amount of the postsynaptic apparatus in contact with the axon terminal. However, the limited size of our samples of NMJs may have reduced the accuracy of these results.

## Discussion

In this study we tested the hypothesis that a systemically delivered protein that retains the signaling function of neural AGRIN would improve the phenotype and NMJ structure in CMS through increasing the numbers of AChR. We found that treatment of mice with mutations in the *Agrn* gene with a modified AGRIN compound (NT1654) restored many aspects of NMJ postsynaptic structure, myofiber morphology and type, and improved muscle strength and body weight. This is the first time that NT1654, a compound that specifically targets the NMJ through protein replacement, has been shown to be effective in a mouse model of CMS. This combined with its potential IV or subcutaneous administration route raises the possibility that NT1654 could be helpful for patients with CMS in the future.

NT1654 is a modified form of mouse AGRIN composed of the c-terminal fragment. We are aware that agrin has a number of binding sites to components of the extracellular matrix such as laminin or heparan sulfate. When engineering NT-1654, solubility of the fragment was one of the key criteria besides resistance to neurotrypsin cleavage. Hence, the portion of the protein that interacts with the matrix was eliminated (y0) and the z8 insert binds to LRP4 only (Hettwer et al., 2014). In addition, the neurotrypsin cleavage β-site has been modified, neurotrypsin being the protease that usually breaks down AGRIN (Stephan et al., 2008). This compound has been shown to cluster AChRs in differentiated C_2_C_12_ cells and *in vivo* in a surgically denervated WT mouse (Hettwer et al., 2014). The authors also tested their compound in a mouse model (SARCO mouse) in which NMJs were denervated through the overexpression of neurotrypsin (Bolliger et al., 2010; Hettwer et al., 2014). Similar to the *Agrn*^nmf380^ mouse, the SARCO mouse had a reduction in body weight and grip strength along with a breakdown in postsynaptic structures. This started to occur around P8 in SARCO mice and a little later in our *Agrn*^nmf380^ mice around P14-18. Like the current study, Hettwer and colleagues also found that NT1654 improved grip strength, recovered postsynaptic structures, and normalized myofiber type in the SARCO mouse (Hettwer et al., 2014). As neurotrypsin cleaves AGRIN it is perhaps not surprising that the two models share similar phenotypes and exhibit similar responses to NT1654.

NT1654 has also been tested in a mouse model of spinal muscular atrophy, where it increased life span, body weight, hindlimb suspension test scores, myofiber size, innervated endplates, and also rescued myofiber type distribution, although without significant increase in spinal motor neurons (Boido et al., 2018). In a rat model of myasthenia gravis, NT1654 improved weight gain, reduced the decrement seen with repetitive nerve stimuli, increased AChRs and their overlap with nerve terminals, and reversed the reduction in myofiber size. The authors suggested that it may be useful as an adjunctive therapy with current treatments for autoimmune myasthenia gravis (Li et al., 2018). In a zebrafish model of presynaptic CMS, the compound was also found to increase spontaneous chorion movements, swimming speed and distance, and AChR cluster intensity (O'Connor et al., 2018).

We started treatment in the first week of life of these animals. This is a time when the axon terminal is very small with an irregular structure and the postsynaptic apparatus is oval with uniform α-bungarotoxin staining, indicating an immature AChR pattern (Slater, 1982). During this time we did not observe any functional differences in body weight or strength between our animals. It was during the initial stages of NMJ maturation that we started to note a difference in body weight and grip strength between WT and Veh animals. It is during this intermediate stage that the NMJs start to show small areas where α-bungarotoxin labelling is not as intense suggesting a more complex pattern, before fully maturing between days P17-21 (Slater, 1982). While the Veh animals did harbor some NMJs that reached maturity, as evidenced by the presence of postsynaptic folds in EM images, it would appear that it is this maturation stage, heavily disrupted in Veh animals, that is impacted with NT1654 administration.

Treatment with NT1654 improved many aspects of NMJ morphology that were disrupted in Veh animals. We observed improvements in both AChR and endplate area in NT animals, as well as an increase in the nerve terminal area—despite this not being found as impacted in Veh animals. Many aspects of structure are associated with function, variation in the frequency of miniature endplate potentials and quantal content has previously been associated with structural size changes in the endplate and nerve terminal in rodents and humans (Hutchinson et al., 1993; Slater et al., 2006; Jones et al., 2016). While it is likely that the structural changes we observed in the current study influenced the function of the NMJ, we would need to perform detailed electrophysiological investigations to confirm this, for example electromyographs or *ex vivo* muscle force experiments. Interestingly we did not observe any changes in the degree of fragmentation in our Veh animals, a phenomenon often observed in animal models of NMJ dysfunction (Hettwer et al., 2014) and aging (Willadt et al., 2016). Although the functional relevance of these fragmented junctions is under debate (Willadt et al., 2016) and their absence in our model does not preclude a functional impairment.

We observed a significant growth in myofibers in the mice treated with NT1654 which we hypothesize is attributable to the normalization of the NMJs. However, AGRIN has been shown to fulfil roles outside of AChR clustering, for example it is involved in skeletal muscle differentiation through its action on excitation-contraction coupling and the development of the membrane resting potential (Jurdana et al., 2009). We cannot, therefore, rule out the possibility that NT1654 caused the hypertrophy of muscle fibers through an additional pathway. Some of these hypertrophic fibers were larger than those observed in WT animals. Occasionally, large fibers are found in neuropathies. However, for denervation to play a role in our study, we would have expected increased numbers of MHC co-expressing and rounded fibers in NT animals. It was previously shown that NT1654 also increases the number of myofibers (Hettwer et al., 2014; Boido et al., 2018), however we did not measure this parameter. In CMS (Huze et al., 2009) and many other neuromuscular disorders (Ciciliot et al., 2013) a fast-to-slow myofiber type change has been noted. In this case the prevention of the TII myofiber atrophy prevented the fast-to-slow myofiber percentage changes observed in *Agrn*^nmf380^ animals (Bogdanik and Burgess, 2011). We hypothesize that these improvements in muscle fiber size and MHC normalization resulted in an increase in muscle and body weight and accounted for the increase in grip strength.

There has been a consistent improvement in NMJ characteristics across species and diseases with the administration of NT1654. The common improvement in AChR clustering would appear to support the theory that NT1654 targets the NMJ through the stimulation of the AGRIN/LRP4/MuSK pathway. As NT1654 primarily targets a postsynaptic pathway, its effects on the presynaptic NMJ structure in this study may initially be surprising with NT1654 increasing the nerve terminal area and complexity of the presynaptic structures. However, the involvement of the AGRIN/LRP4/MuSK pathway in controlling presynaptic differentiation is well known. The *Agrn*^nmf380^ mouse demonstrates abnormal presynaptic sprouting (Bogdanik and Burgess, 2011), and expression of a mini-agrin compound in skeletal muscle can restore presynaptic terminals in AGRIN deficient mice (Lin et al., 2008). Molecules downstream of AGRIN also impact the presynaptic terminal, with presynaptic NMJ functional and structural abnormalities being noted in mice with antibodies against LRP4 (Shen et al., 2013) and MuSK (Viegas et al., 2012). This strongly supports the presence of bi-directional signaling between the pre and postsynapse and could explain the benefits on presynaptic structures that we observed with NT1654 administration. It will be interesting to assess if NT1654 is effective in CMS subtypes caused by presynaptic (Herrmann et al., 2014; O'Connor et al., 2016) or even glycosylation related mutations (Senderek et al., 2011; Cossins et al., 2013). A number of neuropathies have also been demonstrated to have NMJ involvement (Sleigh et al., 2014; Cipriani et al., 2018), for which NT1654 may offer a therapeutic potential.

These findings are promising as currently treatment options for patients with CMS are limited and very much depend on the nature of the gene mutation (McMacken et al., 2017). Acetylcholinesterase (AChE) inhibitors, for example pyridostigmine, work by blocking the enzyme that breaks down the acetylcholine bound to the AChR on the postsynaptic membrane, thereby prolonging the open state of the receptors. However, in patients who have mutations resulting in AChE deficit (COLQ) and with mutations in DOK7 this treatment is detrimental and should be avoided. Some patients respond to 3,4-Diaminopyridine which blocks presynaptic potassium channels, thus prolonging the release of ACh into the synaptic cleft. However, this can be accompanied by dose-related side effects. Sympathomimetics, for example salbutamol and ephedrine, have been successfully used to treat certain CMS. Their exact mechanism of action is currently unknown, but they have been shown to act postsynaptically (Clausen et al., 2018; McMacken et al., 2018, 2019; Webster et al., 2020). However, they can take a while to provide clinical improvement and again can have side effects, including cardiac complications. For these reasons it is important to find other therapeutics that can replace or even work in conjunction with already established treatments.

In this study, animals were treated daily for 30 days, starting at P5 with a subcutaneous injection of the compound. Previous investigations have shown these mice to start developing NMJ dysfunction between P9 and P13 (Bogdanik and Burgess, 2011). While we would have preferred to begin treatment of the *Agrn*^nmf380^ mice after the onset of NMJ pathology and carry it on for longer than 30 days to test if the drug could reverse rather than prevent the pathology, the severe phenotype of the animals limited the duration of experiments. Despite this limitation we were still able to detect changes with this shortened protocol. In addition, we originally aimed to examine both male and female mice, however, *Agrn*^nmf380^ mice were not born in sufficient numbers to provide adequate numbers of animals in each group. This was made more difficult by sex-related differences, for example male WT animals consistently had larger values than females in aspects of NMJ morphology like AChR area endplate perimeter. It would be interesting to see if these sex-related differences influenced how males and females responded to treatment. This study examined functionally very different muscles during testing. Our *in vitro* investigations focused on hindlimb muscles, however, given the differences noted between WT and Veh animals in forelimb grip strength and the improvements noted following drug treatment, it would be interesting to perform *in vitro* investigations on selected muscles of the forelimb in future. The underlying structural differences and responses to pathology in NMJs of different regions in the mouse (Sleigh et al., 2014; Jones et al., 2016) could have influenced the responses to NT1654.

In conclusion, treatment of *Agrn*^nmf380^ mice with the modified AGRIN compound (NT1654) resulted in the prevention of pre- and post-synaptic NMJ morphology breakdown, which we hypothesize contributed to myofiber hypertrophy in both TI and TII myofibers, and importantly prevented the atrophy of TII myofibers. In turn this caused an increase in muscle and animal weight and an increase in forelimb strength. This study adds to the evidence of the efficacy of NT1654 in conditions where the NMJ is compromised and strongly suggests that patients with CMS attributable to mutations in *AGRN* could benefit from this treatment.

## Data Availability Statement

The raw data supporting the conclusions of this article will be made available by the authors, without undue reservation.

## Ethics Statement

The animal study was reviewed and approved by Newcastle University Animal Welfare and Ethical Review Body (AWERB).

## Author Contributions

HL, RH, SH, AM, and GM developed the project concept. SS, RH, GM, TD, and KQ performed the experiments and data analysis. HL, RH, AR, CS, and EO provided ongoing supervision for the project. SS wrote the manuscript with input from HL and CS. All authors edited and approved of the submitted version.

## Conflict of Interest

SH and AM are employed by Neurotune AG. Remaining authors declare that the research was conducted in the absence of any commercial or financial relationships that could be construed as a potential conflict of interest
