# Supplementary material for: Modulation of the Acetylcholine Receptor Clustering Pathway Improves Neuromuscular Junction Structure and Muscle Strength in a Mouse Model of Congenital Myasthenic Syndrome
Source: Front Mol Neurosci. 2020 Dec 17;13:594220. doi: 10.3389/fnmol.2020.594220 (PMC7773664; doi:10.3389/fnmol.2020.594220)
Supplement: Supplementary file 1 [file Data_Sheet_1.PDF]

Supplementary Material

Supplementary Figure 1

Supplementary Figure 1: Clustering of AChRs occurs through the AGRIN/LRP4/MuSK pathway. Neural AGRIN expressed by motor nerves, binds to LRP4, which causes phosphorylation of MuSK. MuSK then recruits DOK7, which further stimulates MuSK phosphorylation. This causes Rapsyn to form complexes with AChRs and help insert them into the post-synaptic membrane where they are anchored to the cytoskeleton. AGRIN is cleaved by the serine protease neurotrypsin. The neurotrypsin resistant 44KDa Agrin fragment (NT1654) can also stimulate the AGRIN/LRP4/MuSK pathway through binding to LRP4 receptors, resulting in an increase of AChRs being inserted into the postsynaptic membrane.

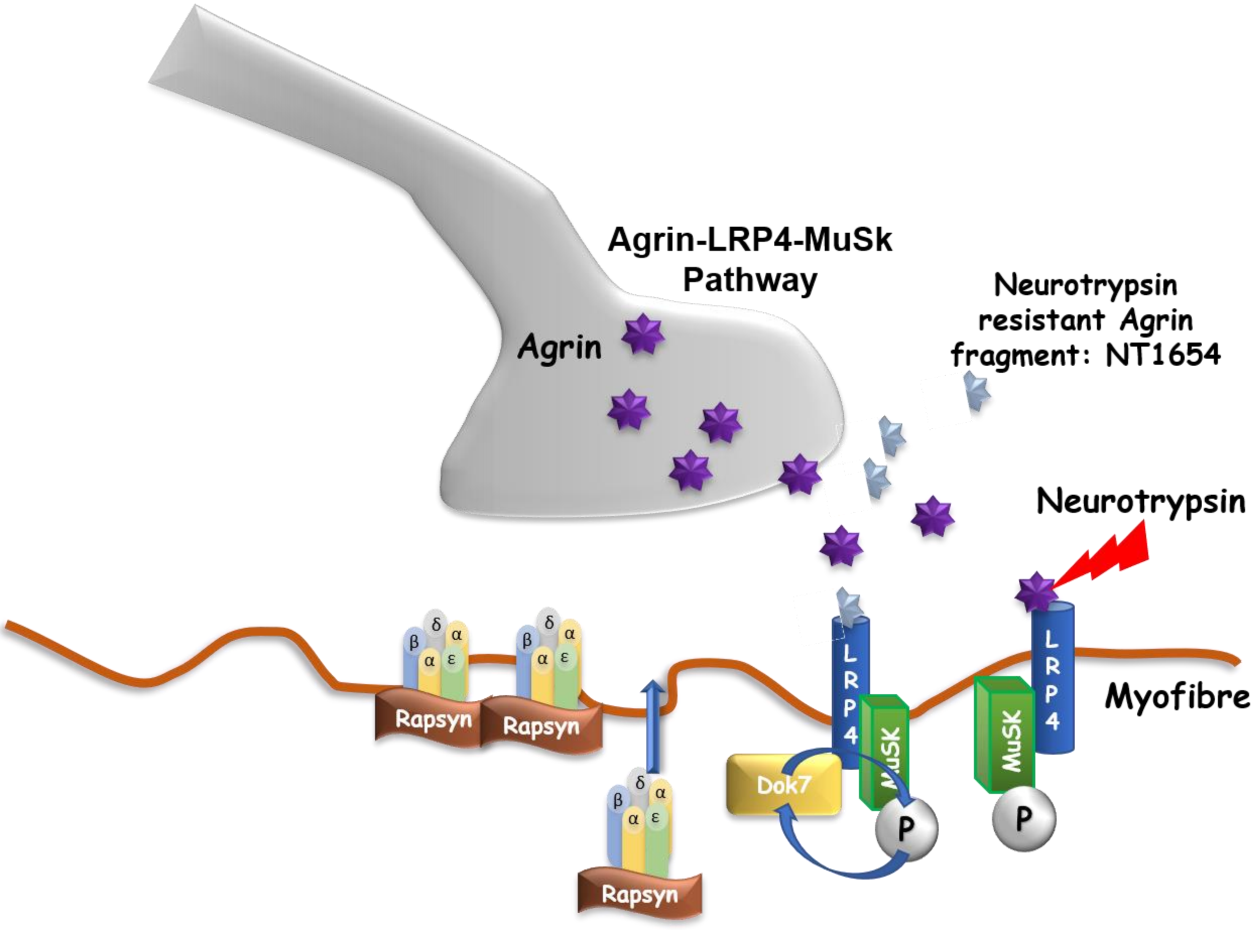

Supplementary Figure 2

Supplementary Figure 2: Analysis of electron microscopy images. Method of analyzing NMJ ultrastructure by EM, accompanied by notes on measurements.

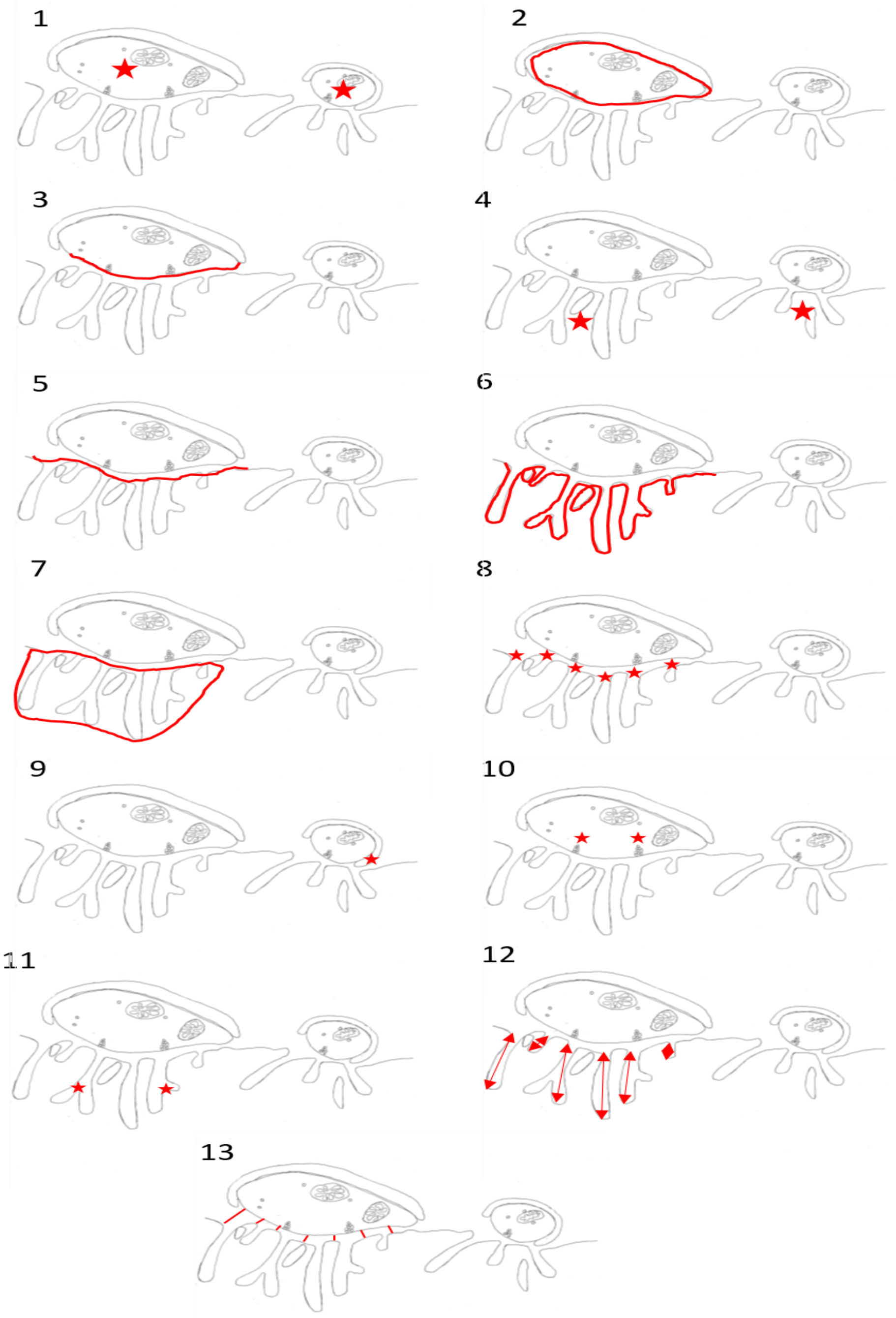

Notes: On some images there will be more than one contact point. This will be reflected in measurement #1 (NT). In this case treat the separate contact points as a different set of measurements, as has been shown here.

1. Number of separate axon terminal profiles (NT): How many terminals can you see in the image?
2. Total area of the axon terminals (NTA): Trace around the terminal.
3. Total length of the axon terminal perimeter in direct contact with the muscle fibre (Prel): Trace the terminal opposite the endplate.
4. Number of distinct regions of postsynaptic folding (FR): Note this is the number of REGIONS, not actual folds.
5. Total length of the subneural apparatus measured along the tops of the folds (SurfL): This does not actually include the folds, you have to trace across those.
6. Total length of postsynaptic membrane including folds (FoldL): Perimeter of muscle folds opposite the terminal. You may need to add a few values together if you have folds that do not have their openings visible on the image.
7. Total area containing the postsynaptic folds (Post A): You need to use a certain amount of judgement for this, as you will draw a shape that encompasses all the folds for that NMJ.
8. Fold openings (NF): Count the openings
9. Invading Schwann cells (ISch): Does the Schwan cell come down into the NMJ?
10. Number of synaptic vesicle clusters opposite folds (SVCl): This is not the number of synaptic vesicles in total but only those that are clustered together AND opposite a fold.
11. Number of branches (NoB): The number of branch points within all the folds.
12. Length of the folds that have openings on to the cleft (CFL): This can probably only be performed on the straighter folds.
13. Width of the synaptic cleft (W_SynC): Measured at the beginning, end, and between folds.

Supplementary Figure 3

Supplementary Figure 3: Body weight of male animals. *Agrn*^{nmf380} male animals were smaller that WT, but administration of NT1654 partially rescued weight in NT animals. Graphs show mean ± S.D, N = 4WT, 3Veh, 2NT.

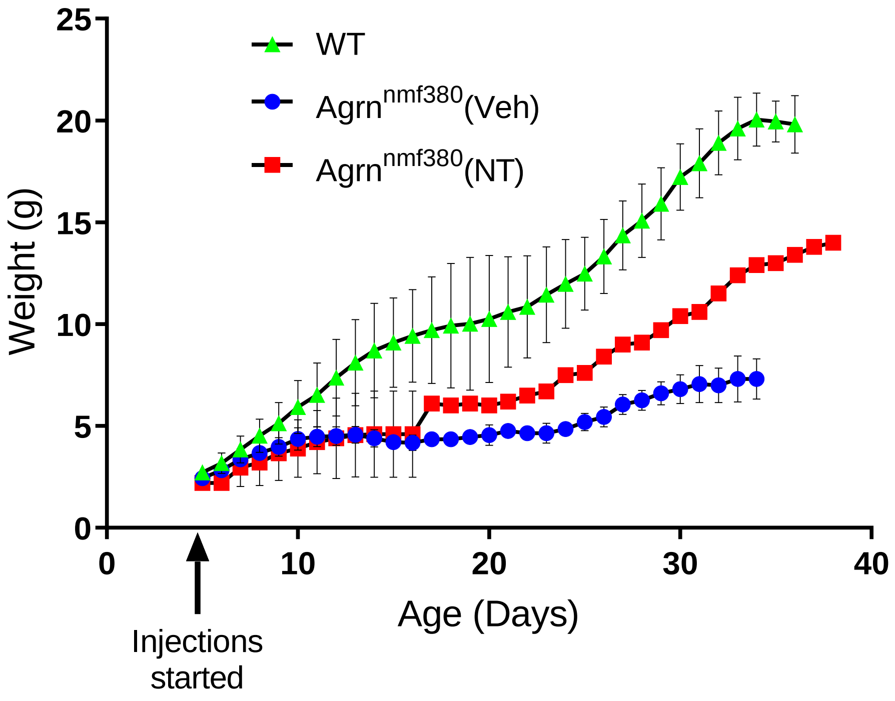

Supplementary Figure 4

Supplementary Figure 4: Electron microscopy investigation of male mice. Intercostal muscle was obtained from male mice and imaged by the Electron Microscopy Research Services at Newcastle University. Arrow heads show postsynaptic folds, arrows show branching, M denotes mitochondria. Scale bar is 500nm.

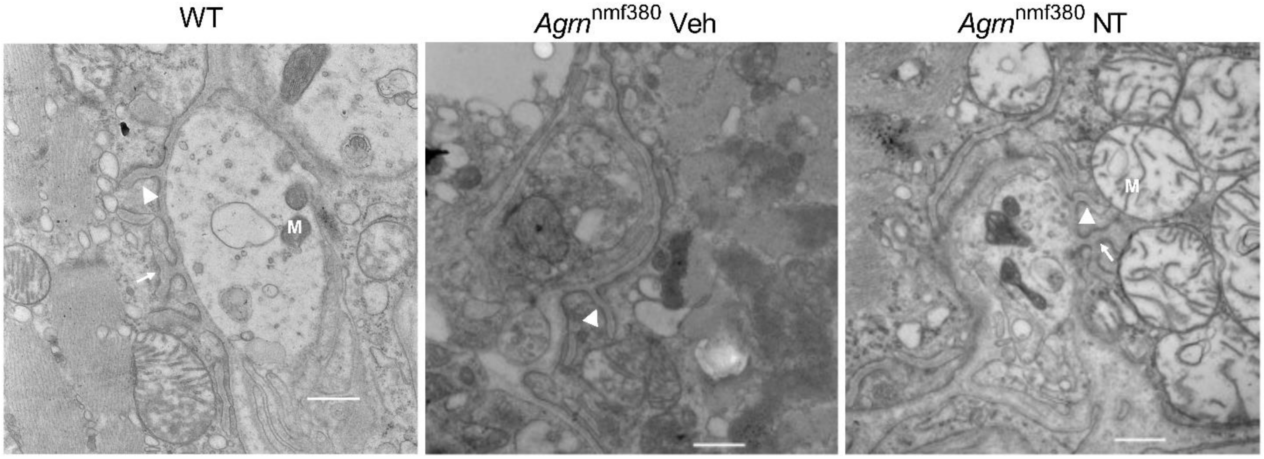

Supplementary Table 1

Supplementary Table 1: Muscle weights of WT, Veh, and NT male and female animals. At the time of harvest muscles were weighed. Data is presented to 2d.p. as mean ± S.D (number of animals). N = 8 WT, 7 Veh, 7 NT.

	WT	$Agrn^{nmf380}$(Veh)	$Agrn^{nmf380}$(NT)
**Quadriceps**	94.25±18.44 (8)	15.429±6.68 (7)	27.14±15.60 (7)
**Gastrocnemius**	77.38±11.31 (8)	14.0±4.73 (6)	21.71 ±12.18 (7)
**Soleus**	5.75±1.91 (8)	1.86±1.46 (7)	3.57±2.94 (7)
**Tibialis Anterior**	29.88±3.04 (8)	7.57±5.19 (7)	15.43±11.15 (7)

Supplementary Table 2

Supplementary Table 2: Fibre Size in WT, Veh, and NT female animals. All values are median (25th percentile, 75th percentile) number of fibres measured. The median value for ‘All Fibre’ types is greater in Veh than NT animals, however this data was analyzed using a Kruskal-Wallis test, which is based on the ranked values. For Veh animals the mean rank was 1771 and for NT animals it was 2001, which resulted in NT animals having a statistically significant increase in fibre size when compared to Veh. N = 4 WT, 3 Veh, 4 NT.

	**WT**	**$Agrn^{nmf380}$(Veh)**	**$Agrn^{nmf380}$(NT)**
All Fibres	857.9 (736.1, 998.8) 2963	590.3 (375, 820.6) 1444	429.9 (361, 968.5) 713
TI Fibres	1030 (919.9, 1158) 825	829.6 (687.5, 1002) 560	513.9 (393.3, 115) 221
Type IIa Fibres	798.1 (695.4, 904.0) 1791	430.2 (313.6, 586) 826	515.9 (346.2, 904) 447
Type IIx Fibres	840.8 (745.7, 984.0) 294	178.6 (132.3, 193.7) 6	375.1 (348.2, 447.3) 42

Supplementary Table 3

Supplementary Table 3: Morphological variables of NMJs in female animals. Initial analysis of NMJ structure in the soleus revealed sex related differences in variables, including nerve terminal perimeter, area, and complexity, AChR area and perimeter, endplate area and perimeter, and overlap. However, NMJ data was only available from 1 Veh and 2 NT males animals so data from 4 WT, 3 Veh, and 5 NT females are presented. Analysis was performed using a previously published method (Jones, Reich et al. 2016), with the addition of two more variables: NMJs with no synaptophysin staining sometimes referred to as 'vacant' and NMJs with no AChR labelled with α-bungarotoxin. From the core variables a number of derived variables were calculated: complexity was calculated using the formula $log10(no.of\ terminal\ branches * no.of\ brnach\ points * total\ length\ of\ branches)$, compactness with the formula $\left(\frac{AChR\ Area}{Endplate\ Area}\right) * 100$, and overlap using $\left(\frac{Total\ Area\ AChR - Unoccupied\ Area\ AChR}{Total\ Area\ AChR}\right) * 100$. All values are to 2d.p. median (25th percentile, 75th percentile) number of NMJs measured. Most variables were not normally distributed and were analyzed using Kruskal-Wallis test followed by Dunn's multiple comparisons test. *Significantly different from WT, $^{\#}$significantly different from Veh. $^{*/\#}$P<0.05, $^{**/\#\#}$P<0.005, $^{***/\#\#\#}$P<0.0005, $^{****/\#\#\#\#}$P<0.0001.

Variable	WT	*Agrn*^{nmf380} Veh	*Agrn*^{nmf380} NT
Core Variables			
Presynaptic			
Absent Synaptophysin staining (%)	8 (0, 18.06) 104	0 (0, 9.09) 31	0 (0, 1.28) 135
Nerve terminal area (µm^2)	51.83 (17.61, 101.5) 96	77.27 (47.56, 121.2) 28	112.5 (65.74, 197.4) 130****
Nerve terminal perimeter (µm)	93.85 (46.6, 134.7) 96	71.54 (52.78, 112.5) 28	120 (74.86, 191.3) 132$^{***\#\#}$
Number of terminal branches	19 (12, 29) 97	7 (4, 11) 28****	15.5 (8, 27.75) 132$^{\#\#}$
Number of branch points	2.52 (0.64, 8) 97	5.5 (3, 10) 28	6.08 (3, 13) 132***
Total length branches	13.6 (5.4, 21.69) 97	15.2 (8.83, 21.28) 28	19.9 (11.2, 32.1) 131***
Postsynaptic			

Absent AChR staining (%)	0 (0, 0) 104	0 (0, 35.29) 31	0 (0, 0) 135
AChR area	159 (91.21, 226) 104	49. 83 (19.47, 114.6) 23****	148.5 (87.39, 249) 133####
AChR perimeter	121.4 (79.86, 170.3) 104	58.26 (41.8, 79.83) 23***	133. 9 (72.79, 221.6) 133####
Endplate area	274.5 (164.1, 425.5)104	104.7 (66.3, 156.5) 23****	329.9 (176.1, 566.6) 133####
Endplate perimeter	72.95 (59.55, 90.25) 104	49.8 (35.18, 65.57) 23***	83.71 (62.08, 112.3) 133####
Endplate diameter	26.52 (22.79, 32.45) 104	19.09 (13.23, 25.43) 21**	29.62 (23.36, 37.75) 133####
Derived Variables			
Presynaptic			
Average length of branches	0.6 (0.3, 1.1) 97	2.15 (1.35, 3.03) 28****	1.4 (0.8, 2.19) 131****#
Complexity	3.27 (2.37, 3.84) 77	2,715 (1.03, 3.4) 28	3.385 (2.58, 3.94) 126#
Postsynaptic			
Average area of AChR clusters	143.1 (80.2, 210.2) 104	40.54 (12.85, 75.78) 23****	117.8 (74.45, 194.7) 132####
Fragmentation	0 (0, 0) 104	0 (0, 0) 23	0 (0, 0) 132
Compactness (%)	54.95 (46.18, 63.93) 104	42.7 (34.9, 56.8) 23**	53.6 (39.45, 63.25) 133
Overlap (%)	24.4 (8, 46.6) 95	36.5 (24.68, 78.6) 22*	51.9 (34.1, 68.05) 129****
Area of synaptic contact	37.66 (9.46, 80.23) 95	16.8 (3.91, 49.19) 22	73.3 (37.24, 145.6) 129 ####****

Associated Nerve Variables			
Axon diameter (µm)	0.72 (0.617, 0.817) 2	1.37 (1.09, 1.68) 14	1.752 (1.27, 2.1) 41*
Number of axonal inputs	1 (1, 1) 3	1 (1, 1) 17	1 (1, 1) 51

Supplementary Table 4

Supplementary Table 4: Analysis of NMJs from male mice using EM. Measurements were made of the nerve terminal area (NTA), length of the presynaptic terminal in contact with the muscle fibre (PreL), number of synaptic vesicle clusters opposite folds (SVCI), total length of the postsynaptic membrane across the tops of the folds (SurfL), total length of postsynaptic membrane including folds (Lfold), the total postsynaptic area containing all the folds (PostA), number of fold opening on the cleft (NF), length of folds opening onto the cleft (CFL), number of branches (NoB), width of the synaptic cleft (W_SynC), the presence of Schwan cells in the synaptic cleft (ISch), and the ratio of the inner to the outer radius of the myelin sheath (G ratio). Many features were derived from these variables: the density of the folds in the endplate Fold Density = NF/PostA, the amount of folded postsynaptic membrane Fold Index = FoldL/SurfL, the number of branches per fold, the bouton area relative to the endplate area, and a measure of the degree of overlap of the pre and post synaptic apparatus Occupancy = SurfL/PreL. All values are median (25th percentile, 75th percentile) number of NMJs measured. While tissue was obtained from 4 WT, 3 Veh, and 3 NT male animals it was only possible to find NMJs in 1 Veh and 1 NT animal. *Significantly different from WT. **P<0.005.

**Variable**	**WT**	***Agrn*^{nmf380} Veh**	***Agrn*^{nmf380} NT**
Core Variable			
Presynaptic			
NTA (µm^2)	3.38 (1.63, 6.32) 33	4.43 (1.23, 6.35) 7	5.02 (3.32, 7.38) 8
PreL (µm)	2.17 (0.83, 3.71) 30	2.93 (1.58, 5.76) 7	2.85 (2.41, 3.58) 8
SVCI	0 (0, 0.5) 33	2 (0, 2) 7	0 (0, 1.75) 8
Postsynaptic			
SurfL (µm)	2.28 (0.9, 4.16) 30	3.14 (1.81, 6.09) 7	3.76 (3.27, 4.91) 8
FoldL (µm)	9.26 (6.73, 18.95) 30	13.33 (10.8, 29.84) 7	17.19 (14.46, 19.8) 8
PostA (µm^2)	88.96 (0.49, 94.91) 29	87.38 (71.61, 93.34) 7	85.65 (63.91, 97.11) 8
NF	4 (1.5, 8) 33	6 (5, 11) 7	6.5 (4.5, 8.75) 8

CFL (µm)	0.57 (0.39, 0.74) 147	0.69 (0.54, 0.87) 52**	0.64 (0.48, 0.71) 53
NoB	1 (0, 2) 33	2 (1, 3) 7	1.5 (1, 2) 8
Other			
W_SynC (µm)	0.08 (0.06, 0.1) 30	0.08 (0.06, 0.09) 7	0.07 (0.06, 0.07) 8
ISch	0 (0, 0) 33	0 (0, 0) 7	0 (0, 0) 8
G ratio	0.83 (0.75, 0.87) 20	0.82 (0.75, 0.94) 15	0.79 (0.78, 0.87) 4
Derived Values			
Fold density	3.39 (2.24, 5.32) 30	2.8 (1.74, 3.57) 7	2.66 (2.01, 3.51) 8
Fold index	4.25 (3.56, 6.90) 30	4.42 (4.07, 6.56) 7	4.33 (2.93, 5.09) 8
Branches per fold	0.18 (0, 0.33) 32	0.27 (0.3, 0.4) 7	0.24 (0.17, 0.28) 8
Endplate area: Bouton area	0.30 (0.49, 1.14) 7	0.72 (0.49, 1.14) 7	0.57 (0.26, 0.75) 8
Occupancy %	93.44 (91.08, 95.99) 14	87.38 (71.61, 93.34) 7	85.65 (63.91, 97.11) 8
